# Photopic negative response recorded with RET*eval* system in eyes with optic nerve disorders

**DOI:** 10.1038/s41598-022-12971-2

**Published:** 2022-05-31

**Authors:** Tsutomu Yamashita, Kumiko Kato, Mineo Kondo, Atsushi Miki, Syunsuke Araki, Katsutoshi Goto, Yoshiaki Ieki, Junichi Kiryu

**Affiliations:** 1 Department of Orthoptics, Faculty of Rehabilitation, Kawasaki University of Medical Welfare, 288 Matsushima, Kurashiki, Okayama 701-0193 Japan; 2 Department of Ophthalmology, Kawasaki Medical School, 577 Matsushima, Kurashiki, Okayama 701-0192 Japan; 3 Department of Ophthalmology, Mie University Graduate School of Medicine, 2-174 Edobashi, Tsu, Mie 514-8507 Japan

**Keywords:** Optic nerve diseases, Electrodiagnosis

## Abstract

Electroretinography (ERG) is used to evaluate the physiological status of the retina and optic nerve. The purpose of this study was to determine the usefulness of ERGs recorded with the RET*eval* system in diagnosing optic nerve diseases. Forty-eight patients with optic nerve disorders, including optic neuritis, ischemic optic neuropathy, traumatic optic neuropathy, and dominant optic atrophy, and 36 normal control subjects were studied. The amplitudes of the photopic negative response (PhNR) were recorded with the RET*eval* system without mydriasis. The circumpapillary retinal nerve fiber layer thickness (cpRNFLT) was determined by optical coherence tomography (OCT). The significance of the correlations between the PhNR and cpRNFLT parameters were determined, and the receiver operating curve (ROC) analyses were performed for the PhNR and cpRNFLT. Patients with optic nerve disorders had significantly smaller PhNRs compared to the control subjects (*P* = 0.001). The ROC analyses indicated that both PhNR and cpRNFLT had comparable diagnostic abilities of detecting optic nerve disorders with PhNR at 0.857 and cpRNFLT at 0.764. The PhNR components recorded with the RET*eval* system have comparable diagnostic abilities as the cpRNFLT in diagnosing optic nerve disorders.

## Introduction

The photopic negative response (PhNR) is a component of the photopic full-field electroretinogram (ERG), and it is a slow negative wave that follows the b-wave. It originates primarily from the retinal ganglion cells and their axons^1–3^. The amplitude of the PhNR is reduced in disorders that affect the innermost retina^4^ and disorders of retinal ganglion cell axon or glial cell. The results of earlier studies have shown that it is reduced in glaucoma^5,6^, retinal vascular diseases^7^, and optic nerve atrophy^8–10^. Thus, the PhNR has been used to help in the diagnosis of retinal nerve fiber disorders. However, recording and analyzing the PhNR using conventional ERG recording systems can be time consuming and the examination procedures are somewhat invasive.

The recently developed handheld ERG recording system, RET*eval* (LKC Technologies Inc., Gaithersburg, MD, USA), allows clinicians to record ERGs quickly and less invasively. The RET*eval* system uses seal-type skin electrodes which allows the recording of ERGs from adults and children relatively simply. It has been reported that the RET*eval* system was as helpful as the conventional ERG recording systems in diagnosing and evaluating ophthalmic diseases such as diabetic retinopathy^11–13^ and retinal vein occlusions^14,15^. The RET*eval* system can record not only the standard full-field ERGs conforming to the International Society for Clinical Electrophysiology of Vision (ISCEV) guidelines but also the PhNRs^16,17^. Although the reliability of the PhNRs recorded with skin electrodes is a concern for many doctors, it has been reported that PhNRs recorded with skin electrodes are as useful in diagnosing diseases as those recorded with the contact lens-type of electrodes^16^.

Gotoh et al. studied the correlation between the PhNR recorded with a conventional ERG recording system and retinal nerve fiber layer thickness (RNFLT) measured by optical coherence tomography (OCT) in 10 patients with optic nerve atrophy^8^. They reported that the two parameters were highly correlated, and they stated that the PhNR can be used to evaluate the function of retinal ganglion cells and their axons in eyes with optic nerve atrophy. However, there has been only one study that determined the correlations between the PhNR and the RNFLT in the subacute phase of optic neuritis (ON)^9^, and there have been no studies evaluating the correlations between the PhNR and the circumpapillary RNFLT (cpRNFLT) in the acute phase of ischemic optic neuropathy (ION). In addition, there have not been any studies that determined whether the PhNR recorded by the RET*eval* system can be used to diagnose optic nerve disorders.

Thus, the purpose of this study was to determine whether PhNRs recorded with the RET*eval* system and cpRNFLT obtained by OCT are significantly correlated in eyes with optic nerve disorders, and to determine whether PhNRs can be used in diagnosing optic nerve disorders.

## Results

### Clinical characteristic of subjects

The demographics of the patients are shown in Table 1. Forty-eight eyes of 48 patients with optic nerve disorders were classified according to the etiology of the disorder. There were 17 eyes with inflammation (acute phase, 6 eyes; chronic phase, 11 eyes). Seven of the 11 patients at the chronic phase received steroid pulse therapy. There were 19 eyes with ischemia with 7 at the acute phase and 12 eyes at the chronic phase. There were 12 eyes with optic atrophy consisting of 6 eyes with traumatic optic neuropathy (TON) and 6 eyes with dominant optic atrophy (DOA). The 13 eyes with ON and ION were placed in the acute ON/ION group and 23 eyes were placed in the chronic ON/ION group.

**Table 1:** Demographics of subjects.

	Control	Overall optic nerve diseases	Acute ON/ION	Chronic ON/ION	TON/DOA	P-value
Subject (eyes)	36	48	10	26	12	
Age (years)	59.7 ± 18.2	55.7 ± 19.1	58.4 ± 16.7	61.4 ± 14.6	41.3 ± 23.2	0.135
Sex (men/women)	14/22	21/27	4/6	8/18	9/3	0.144
Refractive error (D)	− 0.88 ± 2.69	− 0.61 ± 2.13	0.33 ± 1.94	0.68 ± 0.54	− 1.50 ± 1.86	0.230

Optic nerve disease group were statistically compared with the control group.

ON, optic neuritis; ION, ischemic optic neuropathy; TON, traumatic optic neuropathy; DOA, dominant optic atrophy.

Data are shown as the mean ± standard deviation.

The mean interval from the onset of the symptoms and signs of optic nerve disorder to the initial examination was 0.68 ± 0.62 months (range, 0–2.3 months) for the acute ON and ION group and 6.63 ± 6.84 months (range, 3.0–32.3 months) for the chronic ON and ION group. The mean interval from the onset of the symptoms to the initial examination was 3.4 years for the TON (range, 3.0 months to 8.6 years).

The mean age of all the patients with optic nerve disorders was 55.7 ± 19.1 years (range, 9 to 87 years), and 21 were men and 27 were women. The data of 36 eyes of 36 of age-matched normal subjects were analyzed in the same way and served as controls. The mean age of the control subjects was 59.7 ± 18.2 years (range, 26 to 87 years) and included 14 men and 22 women. There was no significant difference in the age and sex distribution between the patients with optic nerve disorders and controls (*P* = 0.135, *P* = 0.144, respectively).

The characteristics of the amplitudes of P_72_, P_min_, P_ratio_, and W_ratio_ of the PhNR, and the a- and b-waves of the ERGs of the eyes with optic nerve disorders and the normal control group are presented in Table 2. In the optic nerve disorders groups, the PhNRs were significantly smaller than that of the normal control group. The a-wave amplitude was significantly larger in the acute ON and ION group than the normal control group (*P* = 0.043). There was no significant difference in the amplitudes of the b-wave between the normal control group and optic nerve disorders group. The averaged pupil diameter during the PhNR recording was significantly larger in the chronic ON and ION groups and TON/DOA groups.

**Table 2:** ERGs and OCT for each group.

	Control	Overall optic nerve disorders	Acute ON/ION	Chronic ON/ION	TON/DOA
a-wave amplitude (μV)	− 3.75 ± 1.93	− 4.81 ± 2.47	− 5.76 ± 2.71	− 4.73 ± 2.09	− 3.95 ± 2.75
*P*-value		0.177	0.043*	0.315	1.000
b-wave amplitude (μV)	19.44 ± 5.89	21.10 ± 7.08	24.05 ± 7.54	21.26 ± 7.03	17.62 ± 5.45
*P*-value		0.724	0.186	0.784	0.894
P_72_ (μV)	− 4.43 ± 2.28	− 1.57 ± 2.43	− 2.34 ± 2.16	− 1.62 ± 2.38	− 0.64 ± 2.68
*P*-value		0.001*	0.047*	0.001*	0.002*
P_min_ (μV)	− 5.35 ± 2.30	− 2.70 ± 2.50	− 3.67 ± 3.00	− 2.41 ± 2.26	− 2.22 ± 2.25
*P*-value		0.001*	0.153	0.001*	0.002*
P_ratio_	0.30 ± 0.15	0.09 ± 0.16	0.14 ± 0.13	0.09 ± 0.14	0.05 ± 0.21
*P*-value		0.001*	0.010*	0.001*	0.002*
W_ratio_	1.09 ± 0.14	0.90 ± 0.11	0.92 ± 0.13	0.88 ± 0.09	0.91 ± 0.12
*P*-value		0.001*	0.004*	0.001*	0.003*
Pupil diameter (mm)	2.04 ± 0.26	2.52 ± 0.78	2.38 ± 0.46	2.82 ± 0.61	3.04 ± 1.27
*P*-value		0.007*	0.213	0.027*	0.048*
cpRNFLT (μm)	102.7 ± 9.9	91.8 ± 46.7	151.4 ± 51.2	76.0 ± 12.2	69.5 ± 11.6
*P*-value		0.001*	0.009*	0.001*	0.001*
GCCT (μm)	92.7 ± 6.6	73.8 ± 14.0	91.1 ± 10.6	70.8 ± 9.9	66.3 ± 6.6
*P*-value		0.001*	0.905	0.001*	0.001*

cpRNFLT, circumpapillary retinal nerve fiber layer thickness; GCCT, ganglion cell complex thickness; ON, optic neuritis; ION, ischemic optic neuropathy; TON, traumatic optic neuropathy; DOA, dominant optic atrophy.

Optic nerve disease groups were statistically compared with the control group. *P < 0.05 was considered significant.

Data are shown as the mean ± standard deviation.

The cpRNFLT was reduced significantly in the patients with overall optic nerve disorders compared to that of the normal control group (*P* = 0.001), and the cpRNFLT was significantly thicker in the acute ON/ION group (*P* = 0.009). The GCCT was reduced significantly in the chronic ON and ION groups and in the TON and DOA groups (*P* = 0.001), but there was no significant difference in patients with acute ON and ION (*P* = 0.905).

**Figure 1:**
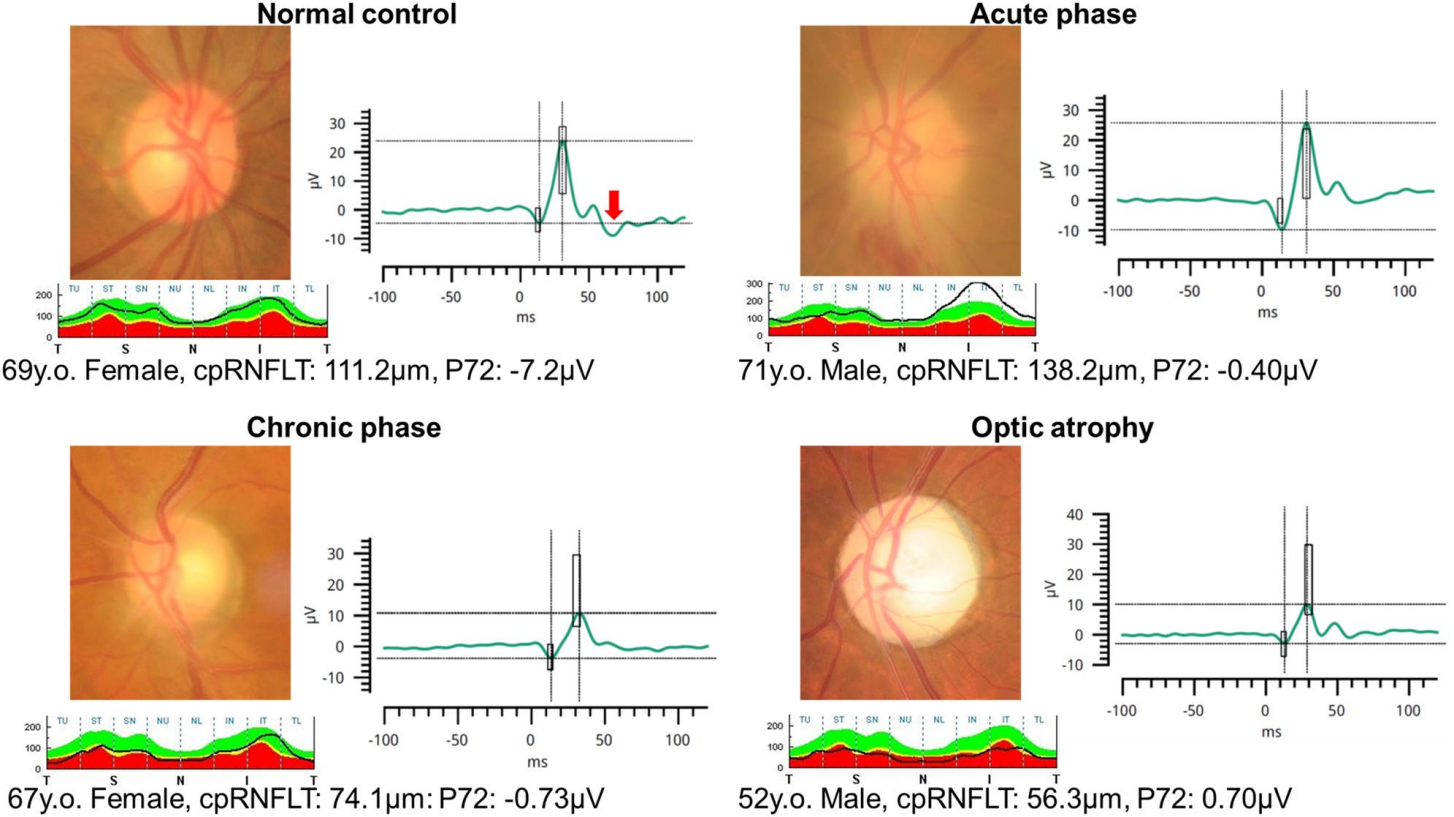
Representative cases of optic nerve disorders and control subjects. In each case, a photograph of the optic nerve papilla, temporal-superior-nasal-inferior-temporal (TSNIT) graph of the circumpapillary retinal nerve fiber thickness (cpRNFLT) and PhNR waveform are shown. The solid black line in the TSNIT graph represents the patient's cpRNFLT. Top left: Normal control subjects. Full-field ERGs showing negative wave immediately after the b-wave (red arrow). Top right: Acute phase of non-arteritic ischemic optic neuropathy. Fundus photograph shows segmental optic disc swelling with flame-shaped hemorrhage. The PhNR is severely attenuated. Bottom left: Chronic phase of non-arteritic ischemic optic neuropathy. Fundus photograph shows mild atrophy of the optic disk. PhNR is severely attenuated. Bottom right: Traumatic optic neuropathy. Fundus photograph shows pallor of the optic disk. The PhNR is severely attenuated.

Fundus photographs and PhNRs of representative cases of optic nerve disorders and control group are shown in Fig. 1. In the control group, full-field ERGs showed a negative wave at 72 ms (red arrow), but in the optic nerve disorders groups, the negative wave after the b-wave was considerably smaller.

### Correlation between PhNRs and cpRNFLT and ganglion cell complex thickness in eyes with optic nerve disorders

The correlations between the different components of the PhNRs and the cpRNFLT and the ganglion cell complex thickness (GCCT) are shown in Table 3. The cpRNFLT was weakly but significantly correlated with the P_72_, P_min_, and P_ratio_ amplitudes in all cases of optic nerve disorders (*r* = − 0.356, *P* = 0.013; *r* = − 0.334, *P* = 0.020; and *r* = 0.299, *P* = 0.039, respectively). In the acute ON/ION eyes, the cpRNFLT was weakly but significantly correlated with the W_ratio_ (*r* = − 0.553, *P* = 0.049), but the cpRNFLT was not significantly correlated with the PhNR components in the chronic ON/ION eyes. In the TON/DOA group, the cpRNFLT was significantly correlated with the P_min_ and the W_ratio_ (*r* = − 0.720, *P* = 0.008; *r* = 0.650, *P* = 0.022; respectively). The correlation between the PhNRs and the GCCT was not significant.

**Table 3:** Univariate analysis between PhNRs and cpRNFLT, GCCT in optic nerve disorders.

Variables	Variables	Overall optic nerve disorders		Acute ON/ION		Chronic ON/ION		TON/DOA	
		*r*	*P*-value	*r*	*P*-value	r	*P*-value	r	*P*-value
cpRNFLT	P_72_	− 0.356	0.013*	0.621	0.024*	− 0.376	0.077	− 0.336	0.286
	P_min_	− 0.334	0.020*	0.473	0.103	− 0.324	0.131	− 0.720	0.008*
	P_ratio_	0.299	0.039*	− 0.566	0.044*	0.228	0.295	0.455	0.138
	W_ratio_	0.170	0.247	− 0.553	0.049*	0.168	0.443	0.650	0.022*
GCCT	P_72_	− 0.194	0.187	0.401	0.174	− 0.044	0.843	− 0.077	0.812
	P_min_	− 0.134	0.365	0.247	0.415	0.027	0.601	− 0.266	0.404
	P_ratio_	0.119	0.419	− 0.324	0.280	− 0.115	0.902	0.056	0.863
	W_ratio_	0.005	0.975	− 0.374	0.208	− 0.042	0.849	0.070	0.829

cpRNFLT, circumpapillary retinal nerve fiber layer thickness; GCCT, ganglion cell complex thickness; ON, optic neuritis; ION, ischemic optic neuropathy; TON, traumatic optic neuropathy; DOA, dominant optic atrophy.

*P < 0.05 was considered significant.

**Figure 2:**
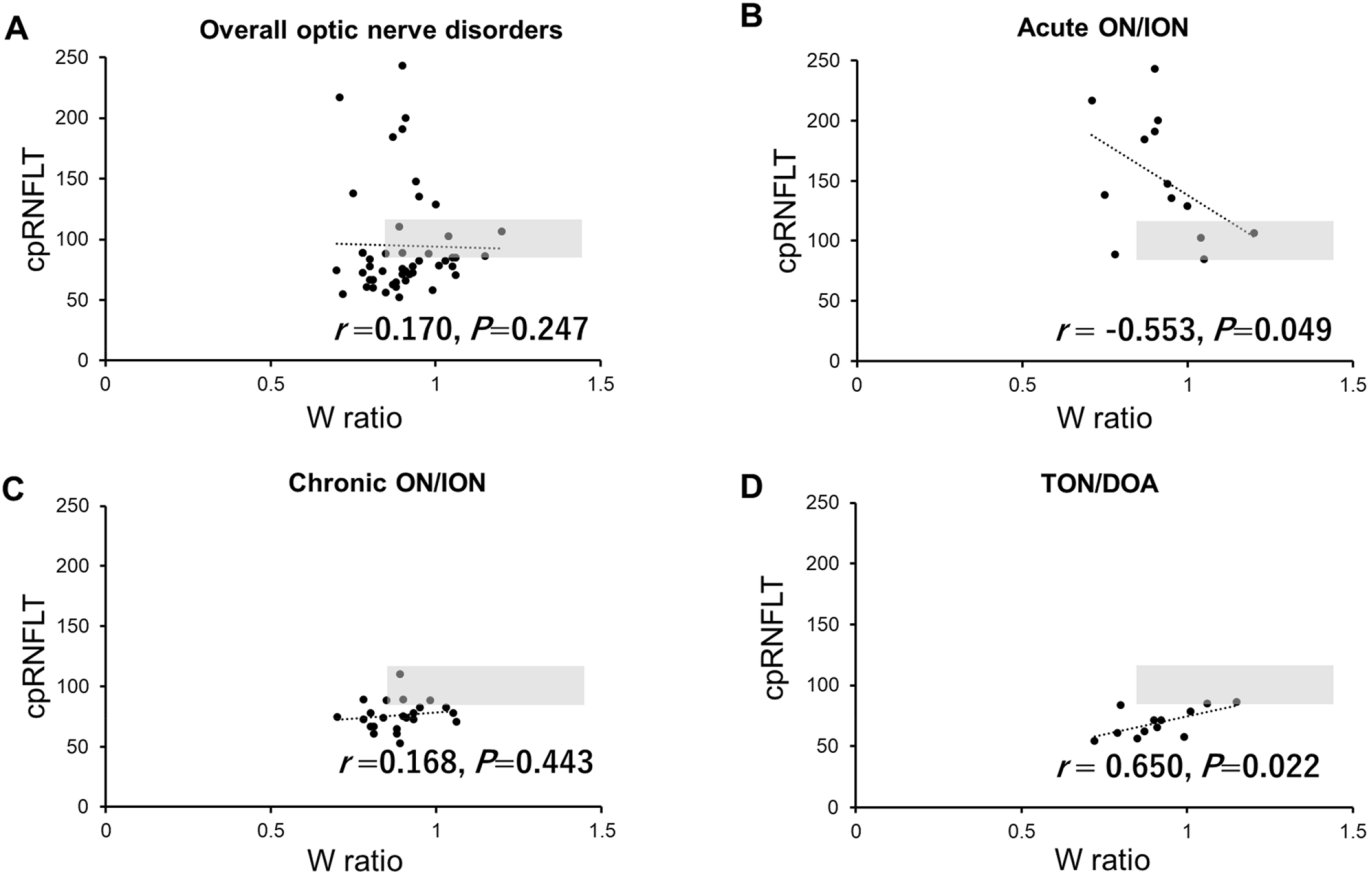
Graph showing the relationship between the W_ratio_ and cpRNFLT in optic nerve disorders. Scatterplots of all of the cases of optic nerve disorders (**A**), acute phase of optic neuritis, and ischemic optic neuropathy (**B**), chronic phase of optic neuritis or ischemic optic neuropathy (**C**), dominant optic neuropathy or traumatic optic atrophy versus cpRNFLT (**D**). The gray areas in the scatterplot show the distribution of data for normal subjects.

A scatter plot of the W_ratio_ and the cpRNFLT is shown in Fig. 2. There was no significant correlation between the W_ratio_ and cpRNFLT for the optic nerve disorders grouped together (*r* = 0.170, *P* = 0.247). However, there was a significant negative correlation between the W_ratio_ and cpRNFLT in eyes at the acute ON and ION phase (*r* = − 0.553, *P* = 0.049) but there was no significant correlation in the chronic ON and ION phase (*r* = 0.168; *P* = 0.443). There was a significant positive correlation between the W_ratio_ and cpRNFLT in the TON/DOA group (*r* = 0.650; *P* = 0.022). Scatter plots of all PhNRs and cpRNFLTs were constructed (Supplementary Fig. 1), and we found that the analyses performed using P_72_, P_min_, and P_ratio_ showed the same trend as the analyses performed using W_ratio_.

### Receiver operating characteristic curve analyses of diagnostic performance in optic nerve disorders

**Figure 3:**
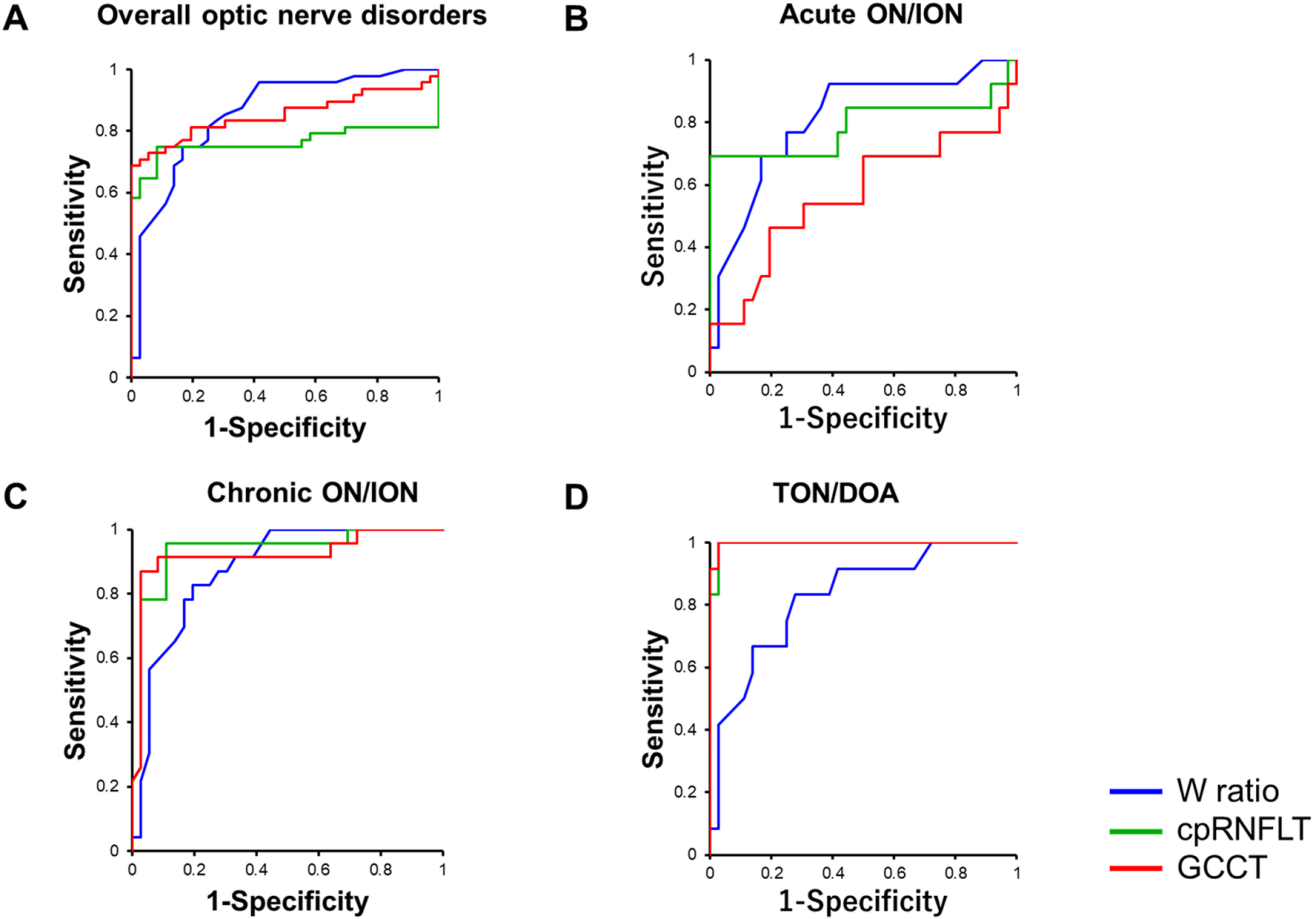
Receiver operating characteristic (ROC) curves for detecting optic nerve disorders. Blue, green, and red lines show the ROC curves for detecting optic nerve disorders using the W_ratio_, cpRNFLT and GCCT as indices respectively. (**A**). Overall optic nerve disorders, (**B**). Acute phase of optic neuritis or ischemic optic neuropathy, (**C**) chronic phase of optic neuritis or optic neuropathy, (**D**) hereditary or traumatic optic atrophy.

We examined whether the different components of the PhNR recorded with the RET*eval* system can be used to differentiate eyes with from the eyes without optic nerve disorders using the receiver operating characteristic (ROC) curve (Fig. 3).

The AUCs for discriminating optic nerve are shown in Table 4. When the AUCs were calculated for all subjects, the AUC was 0.857 for the W_ratio_, 0.764 for the cpRNFLT, and 0.853 for the GCCT. The AUCs for P_72_ was 0.790, that for P_min_ was 0.784, and that for the P_ratio_ was 0.833 for all subjects (Table 4.). The AUCs using P_72_, P_min_ and the P_ratio_ were comparable to the AUC using the W_ratio_. The sensitivity and specificity in detecting overall optic nerve disorders were 75% and 83.3% for the W_ratio_ and 75% and 91.7% for the cpRNFLT.

**Table 4:** Comparison of diagnostic abilities of PhNRs and OCT in optic nerve disorders.

	Overall			Control + Acute ON/ION			Control + Chronic ON/ION			Control + TON/DOA		
	AUC	Sensitivity (%)	Specificity (%)	AUC	Sensitivity (%)	Specificity (%)	AUC	Sensitivity (%)	Specificity (%)	AUC	Sensitivity (%)	Specificity (%)
P_72_	0.790	66.7	77.8	0.735	76.9	61.1	0.772	73.9	66.7	0.844	75.0	88.9
P_min_	0.784	72.9	72.2	0.689	61.5	77.8	0.786	73.9	69.4	0.841	75.0	88.9
P_ratio_	0.833	77.1	83.3	0.784	69.2	83.3	0.833	78.3	80.6	0.833	83.3	83.3
W_ratio_	0.857	75.0	83.3	0.810	76.9	75.0	0.877	82.6	80.6	0.829	83.3	72.2
cpRNFLT	0.764	75.0	91.7	0.789	69.2	100	0.935	95.7	88.9	0.995	100	97.2
GCCT	0.853	81.3	80.1	0.567	53.9	69.4	0.920	91.3	91.7	0.998	100	97.2

cpRNFLT, circumpapillary retinal nerve fiber layer thickness; GCCT, ganglion cell complex thickness; AUC, area under the curve; ON, optic neuritis; ION, ischemic optic neuropathy; TON, traumatic optic neuropathy; DOA, dominant optic atrophy.

Next, we analyzed ability of each PhNR component to detect optic nerve disorders by classifying the cases into those with acute ON/ION, chronic ON/ION, and TON/DOA. When the cpRNFLT was used, the diagnostic ability was higher when the optic nerve disorders were classified into each group than when analyzed overall. In addition, the sensitivity and specificity of the diagnosis had higher values. On the other hand, when the W_ratio_ was used, there was no difference in the diagnostic accuracy; the sensitivity and specificity in diagnosing optic nerve disorders regardless of the classification of the patients.

Finally, we performed ROC analysis using the GCCT and found that the diagnostic ability of the GCCT was equal to the cpRNFLT and the W_ratio_ in the chronic ON/ION and TON/DOA eyes, but it was low at 0.567 in the acute ON/ION eyes.

## Discussion

Our results showed that the averaged amplitudes of the different parameters of the PhNR in patients with optic nerve disorders were reduced significantly to 30–80% of that of normal control eyes. The ROC analyses showed that the diagnosis of optic nerve disorders with the different PhNR components was comparable to that of the diagnosing with the cpRNFLT. This suggests that recording PhNRs with the RETeval system may be a useful examination to evaluate retinal ganglion cell function in eyes with optic nerve disorders.

There are a variety of diseases that can cause abnormalities in the retinal ganglion cells including ischemia, degenerative changes, trauma, toxicity, and nutritional abnormalities. Many studies have evaluated the changes of the RNFLT in these diseases using OCT but relatively few studies have used electrophysiological tests, such as visual evoked potentials, pattern ERGs, and PhNR to evaluate functional change, and their clinical significance has not been fully discussed.

The significant correlation between the PhNR and cpRNFLT has been reported in healthy subjects^18^, glaucoma patients^3,19^, and patients with optic nerve atrophy^8^. In our study, the scatter plots of the cpRNFLT and W_ratio_ for all of the optic nerve disorders were not significantly correlated (Fig. 2, upper left), and the P_72_, P_min_, and P_ratio_ had only a weak correlation with the cpRNFLT (Table 3). On the other hand, there was a moderately but significant correlation between PhNR and cpRNFLT in the acute ON/ION and TON/DOA groups. We hypothesized that this may be because the correlations were canceled in the overall optic nerve disorders, including both acute ON/ION, in which PhNR and cpRNFLT were correlated negatively (Fig. 2B), and TON/DOA, in which PhNR and cpRNFLT were correlated positively (Fig. 2D).

The question then arises as to why we did not find a significant correlation between cpRNFLT and W_ratio_ in the eyes with chronic ON/ION while a significant correlation was found in the acute ON/ION. The results showed that the standard deviation of the cpRNFLT in the acute ON/ION was large at 51.2 µm and it was 12.2 µm in the chronic ON/ION (Table 2). Thus, the cpRNFLT had less variability in the chronic phase of ON/ION. We suggest that the lower variability in the cpRNFLT may be the reason for the lack of significant correlations between the cpRNFLT and the W_ratio_ in the chronic phase of ON/ION.

There was a significant positive correlation between the W_ratio_ and cpRNFLT in the eyes with TON/DOA. TON, which can be cause by blunt trauma to the optic nerve, has been reported to lead to a thinning of the cpRNFLT which progresses slowly^20^. DOA is a progressive atrophy of the optic nerve caused by degeneration of the retinal ganglion cells^21^. Studies examining the association between the PhNR and optic atrophy have shown that PhNR is significantly reduced in DOA compared to the normal group^22^, and PhNR and RNFLT are significantly correlated in TON^8^. A high correlation between the W_ratio_ and cpRNFLT in the TON/DOA group was probably due to the thinning of the cpRNFLT and the simultaneous decrease of the W_ratio_ with the degeneration of the retinal ganglion cells.

Our results showed that the correlation between the PhNRs and GCCT was not significant. This may be due to the fact that GCCT changes progress with a time lag from the onset of optic nerve disease, i.e., there was no significant change in GCCT in the acute phase of ON/ION compared to the normal group, and significant thinning of GCCT in the chronic phase of ON/ION and TON/DOA (Table 2). It should also be noted that GCCT is a parameter that reflects the structural changes in the macula, while PhNR is a parameter that represents functional changes in the entire retina. We believe that these can explain why the GCCT was not significantly correlated with the PhNRs.

There have been several studies that evaluated the diagnostic abilities of the PhNR in detecting glaucoma. A study on nonhuman primate of glaucoma reported that the AUC for the diagnostic ability of the PhNR in detecting glaucoma was 0.90^23^. In studies on glaucoma patients, the AUC was reported to be 0.60–0.80^6,24–26^. In our study, the AUC for diagnosing all of the cases of optic nerve disorders was 0.78–0.85 which is comparable to previous reports in glaucoma patients.

Reports on the sensitivity and specificity for the diagnostic abilities of PhNR in glaucoma patients have stated that the sensitivity ranged 53.3–76.7% and the specificity ranged 80–90%^19^. In our study of optic nerve disorders, the sensitivity ranged 61.5–83.3% and the specificity ranged 61.1–88.9%, AUC was highest in the analysis using W_ratio_. The sensitivity tended to be slightly higher than the study in glaucoma patients. We believe that the sensitivity and specificity in this study of optic nerve disorders were not poorer than the results obtained in studies of glaucoma patients.

Then, the question arises as to which is better in diagnosing optic nerve disorders, the cpRNFLT or the PhNR? A study that analyzed the diagnostic performance of PhNR and cpRNFLT in glaucoma patients reported that the diagnostic abilities of these parameters were comparable^19^. Although no study has analyzed the diagnostic abilities of both PhNR and cpRNFLT in patients with optic nerve disorders, a study that analyzed the diagnostic abilities of optic nerve disorders using cpRNFLT reported that the sensitivity and specificity were 72% and 91% respectively^27^. The sensitivity and specificity of the W_ratio_ for diagnosing optic nerve disorder was 75.0% and 83.3% respectively in our study. These values are comparable to that of the earlier study using cpRNFLT^27^. Therefore, we suggest that the diagnostic abilities of PhNR in optic nerve disorders are comparable to that of cpRNFLT. Considering that the diagnostic abilities of PhNR is not inferior to that of cpRNFLT, and the interpretation of PhNR is easier because the PhNR becomes uniformly smaller than that of the normal control subjects regardless of the interval since the onset of optic nerve disorders, we believe that using both the cpRNFLT and PhNR will enable clinicians to diagnose optic nerve disorders more accurately.

The mean pupil diameter during PhNR recordings was significantly larger in the optic nerve diseases except for the acute ON/ION group. Previous studies have reported that degree of impaired pupillary light response in patients with chronic phase of optic neuritis was significantly correlated with the severity of the neurologic disability and RNFLT^28^. We suggest that the larger pupil diameter during PhNR recording in chronic ON/ION and TON/DOA compared to the control group may be due to irreversible impairment of optic nerve function caused by the primary disease.

Then, does the difference in pupil diameter affect PhNR? We have reported the effect of pupil diameter on ERGs when recording flicker ERGs without mydriasis using RETeval system^29^. We found that pupil diameter did not affect the ERG results as long as the pupil diameter did not exceed 6.5 mm. In our present study, the mean pupil diameter during PhNR recordings was less than 6.5 mm, and we believe that differences in pupil diameter did not affect the PhNR.

Finally, the amplitude of a-wave was significantly larger in the acute ON/ION than that of the control group. A study that recorded PhNR from patients with multiple sclerosis who developed ON reported that the amplitude of the a-wave was not significantly different from that of the control group^9^. The increase in the a-wave amplitude may be limited to the very early stages of ON, and it will be necessary to record PhNRs from a larger number of subjects with ON during the course of the disease then determine whether the increased a-wave amplitude of PhNR is significant.

There are several limitations in this study. First, the number of patients in each group was small when classified by stage and etiology. The number of TON and DOA was especially small so that it was difficult to statistically analyze each as a single group so we grouped them together as the TON/DOA group. This small number was because even though optic nerve disorders are relatively common in daily clinical practice, there were few cases that met all of the inclusion criteria. We plan to continue our research and analyze more subjects with optic nerve disorders. The second limitation was that this study was a cross-sectional study, and we did not evaluate the changes in PhNR during the acute and chronic phases in the same patients with ON and ION. We need to perform a longitudinal study on eyes with ON and ION while performing ERGs to use PhNR as diagnostic tool in clinical practice.

In conclusion, the amplitudes of the PhNR recorded with the RET*eval* system are significantly correlated with cpRNFLT in patients with acute phase of ON/ION, and optic atrophy induced by hereditary disease and trauma. We conclude that the different components of the PhNRs are as useful as the OCT for diagnosing optic nerve diseases. PhNR is a parameter that is easy to interpret because it becomes uniformly lower in patients with optic nerve disorders, and we recommend the concomitant use of OCT and PhNR to improve the accuracy of the diagnosis of optic nerve disorders.

## Methods

### Study design

This was a retrospective, single center study conducted at the Kawasaki Medical School Hospital between July 2017 and April 2019. The Medical Ethics Committee of the Kawasaki Medical School Hospital approved the procedures used (No. 3296), and the procedures conformed to the tenets of the Declaration of Helsinki of the World Medical Association. After the nature and possible consequences of the study were explained, a signed written informed consent was obtained from each of the participating patients.

### Participants

The optic nerve disorders were diagnosed by a neuro-ophthalmology specialist (AM) based on the clinical findings. The participants underwent comprehensive ophthalmologic examinations including measurements of the best-corrected visual acuity (BCVA) and intraocular pressure, and slit-lamp biomicroscopy, color fundus photography, optical coherence tomography, and kinetic or static visual field perimetry. Patients were excluded if they had a systemic neurological disease or history of a retinal disease including diabetic or hypertensive retinopathy and a history of eye surgery. In patients with bilateral optic neuropathy, one eye was selected at random for the statistical analyses. In addition, 36 age and sex matched healthy subjects were studied in the same way and served as controls. Only one eye was randomly selected in the control group. The ON and ION were classified into the acute phase and chronic phase according to past reports^30–32^. Acute ON/ION was defined as having a swelling of the optic nerve that was evident ophthalmoscopically and was detected within three months of the onset of symptoms and signs. Chronic ON/ION was defined when more than three months had passed since the onset of symptoms and the swelling of the optic disc had disappeared. Both TON and DOA are diseases that lead to optic atrophy, and eyes with these diseases were placed in the TON/DOA group. The acute ON/ION, the chronic ON/ION, and the TON/DOA groups were collectively referred to as overall optic nerve disorder.

### PhNR recording by RET*eval* system

Full-field PhNRs were recorded with the RET*eval* system from all patients and controls with natural pupils under room lighting. The PhNR recordings were performed by experienced ophthalmic technicians (SA, KG). After cleaning the skin with 70% isopropyl alcohol swabs, the sensor strip skin electrode array (Sensor Strip; LKC Technologies Inc., Gaithersburg, MD, USA) was carefully placed 2 mm below the lower eyelid on the orbital rim of both eyes and connected to the sensor strip lead. This strip included the active, reference, and ground electrodes in a single adhesive tape. The contralateral eye was covered during the recordings. A mini Ganzfeld dome was placed in front of the eye, and the eye was stimulated with red flashes (621 nm, 38 Td·s) on a steady blue background (470 nm, 380 Td) which saturated the rod and isolated the cone responses. The stimulus frequency was 3.4 Hz, and 200 responses were averaged for each recording to increase the signal-to-noise ratio. The patients were instructed to fixate a fixation point within the dome, and the patient's fixation was monitored by an infrared camera. During the stimulation, the pupil size (mm^2^) was automatically measured in real-time, and the stimulus flash luminance (cd-s/m^2^) was continuously adjusted to maintain a constant flash retinal illuminance (Td-s) by the following equation:

$$ \begin{aligned} {\text{Photopic flash retinal illuminance }}\left( {{\text{Td-s}}} \right) & ={\text{Photopic flash luminance }}\left( {{\text{cd-s}}/{\text{m}}^{{2}} } \right)\, \hfill \\ & \quad \times \,{\text{pupillary area }}\left( {{\text{mm}}^{{2}} } \right). \hfill \\ \end{aligned} $$

### Analyses of ERGs

**Figure 4:**
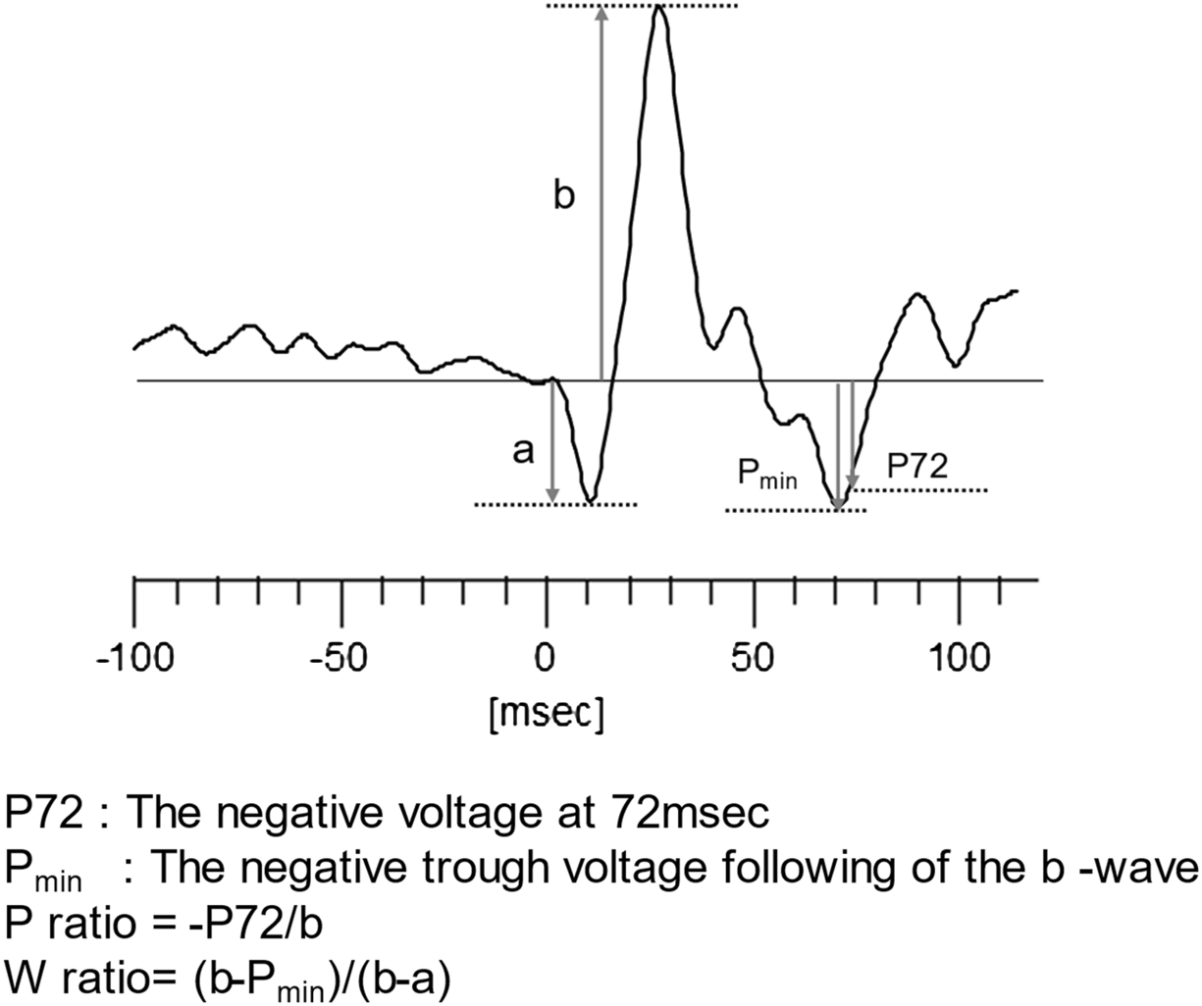
Illustration of the methods to measure various components of the ERG waveform. Arrows represent the amplitude of each parameter. The a-wave amplitude was measured from the pre-stimulus baseline to the first negative trough (a). The b-wave amplitude was measured from the a-wave trough to the positive peak (b-a). The P72 amplitude was measured at 72 ms after the stimulus onset, and the amplitude of P_min_ was measured at the negative trough following the b-wave. The calculation of P_ratio_ and W_ratio_ was done as shown in the figure.

The methods used to measure each ERG component are shown in Fig. 4. The a-wave amplitude was measured from the pre-stimulus baseline to the first negative trough, and the b-wave amplitude was measured from the a-wave trough to the b-wave peak. The PhNR amplitude was measured at two points; at 72 ms from the stimulus onset and the second was measured at the negative trough immediately following the b-wave between 55 and 100 ms. The first amplitude was measured from the pre-stimulus baseline to the negative trough at 72 ms after the stimulus onset (P_72_), and the second amplitude was measured from the baseline to the negative trough after the b-wave (P_min_). The ratio of PhNR amplitude at 72 ms to the b-wave peak was designated as the P_ratio_ (P_72_ voltage/b-wave voltage)^17^. The ratio of the b-wave peak voltage to the PhNR trough voltage to b-wave amplitude was designated as the W_ratio_ (b-wave voltage − Pmin voltage)/(b-wave voltage − a-wave voltage)^33^. We used the W_ratio_ in most of the statistical analyses because it has been reported to have the lowest inter-individual, inter-session, and interocular variations^33^. The PhNR data were analyzed by specialists of visual electrophysiology (MK, KK). Cases were excluded if the ERGs could not be recorded due to significant vision loss or the ERGs could not be analyzed due to significant noise.

### Protocol for OCT examinations

OCT examinations were performed by experienced ophthalmic technicians (SA, KG). The OCT images were recorded with the RTVue-100 SD-OCT (RTVue-100, Optovue, Inc., Fremont, CA, USA), which can acquire 26,000 A-scans/sec and had a 5-μm depth resolution. The Versuib 4.0 software of RTVue-100 was used for data acquisition. The cpRNFLT was determined using the nerve head map 4-mm protocol in the three-dimensional baseline mode in which data along a 3.45-mm diameter circle around the optic disc were recalculated with a map created from en face images that used 6 circular scans ranging from 2.5 to 4.0 mm in diameter (587 or 775 A scans each). The scans were centered on the optic disc and consisted of data from 12 linear radial scans (3.4-mm length, 452 A scans each). This scan protocol recorded 9510 A scans/0.39 s. The cpRNFLT map was generated from the three-dimensional video images after defining the optic disc margin and anchoring points of the retinal pigmented epithelium by the examiners.

The ganglion cell complex (GCC) protocol was used to obtain the macular measurements. This protocol consisted of 1 horizontal line scan of 7 mm in length (467 A scans) and 15 vertical line scans 7 mm in length (each 400 A scans) at 0.5-mm intervals. The center of the GCC scan was shifted 0.75 mm temporally to improve the sampling of the temporal periphery. This scan configuration recorded 14,810 A scans in 0.58 s. The GCC thickness (GCCT) was measured from the inner limiting membrane (ILM) to the outer boundary of the inner plexiform layer (IPL). The built-in software allowed an automated segmentation not only of the total retina from the ILM to the outer border of the retinal pigment epithelium but also of the outer retina from the inner border of the IPL to the outer border of the retinal pigment epithelium.

The recording of SD-OCT images was done on the same day as the PhNR recordings. The SD-OCT data were analyzed by 2 neuro-ophthalmology specialists (AM, TY). The images of the SD-OCT that had a signal strength index (SSI) less than 45 were excluded and images were also excluded if they included involuntary saccades or blinking artefacts, when an algorithm segmentation error was present, or when the retinal layers were poorly imaged.

### Statistical analyses

Kruskal Wallis tests were used to compare the control group to the optic nerve diseases groups for patient background factors, PhNRs, and OCT. We used the Steel–Dwass method for multiple comparisons, and the significance levels were adjusted using the Bonferroni method. The receiver operating characteristic (ROC) curves were created for the W_ratio_, cpRNFLT, and GCCT in the patients with optic nerve disorders to investigate the ability of these parameters to differentiate eyes with optic nerve disorders from normal control eyes. An ROC curve is a plot of the true-positive rate versus the false-positive rate for all possible cutoff points. The area under the ROC curve (AUC) was used to determine the diagnostic accuracy of each parameter.

All of the statistical analyses were performed using the IBM SPSS Statistics software program (version 23.0, SPSS Japan, Inc, Tokyo, Japan). Differences in diagnostic ability (AUC) was tested for statistical significance using MedCalc statistical software version 18.6 (MedCalc Software Inc, Mariakerke, Belgium). The data are presented as the mean ± SD, and *P* values of < 0.05 were taken to be statistically significant.

### Statement of human rights

All procedure performed in this study involving human participants were in accordance with ethical standards of the institutional and/or national research committee and with the 1964 Helsinki declaration and its later amendments or comparable ethical standards.

### Statement on the welfare of animals

No animals were used in this study.

## Supplementary Information

Supplementary Figure 1.

## Data Availability

The datasets used and/or analyzed during the current study are available from the corresponding author on reasonable request.
