# Supplementary material for: Photopic negative response recorded with RETeval system in eyes with optic nerve disorders
Source: Sci Rep. 2022 May 31;12:9091. doi: 10.1038/s41598-022-12971-2 (PMC9156775; doi:10.1038/s41598-022-12971-2)
Supplement: Supplementary file 1 — Supplementary Figure 1. [file 41598_2022_12971_MOESM1_ESM.docx]

**Supplementary figure 1.**

**
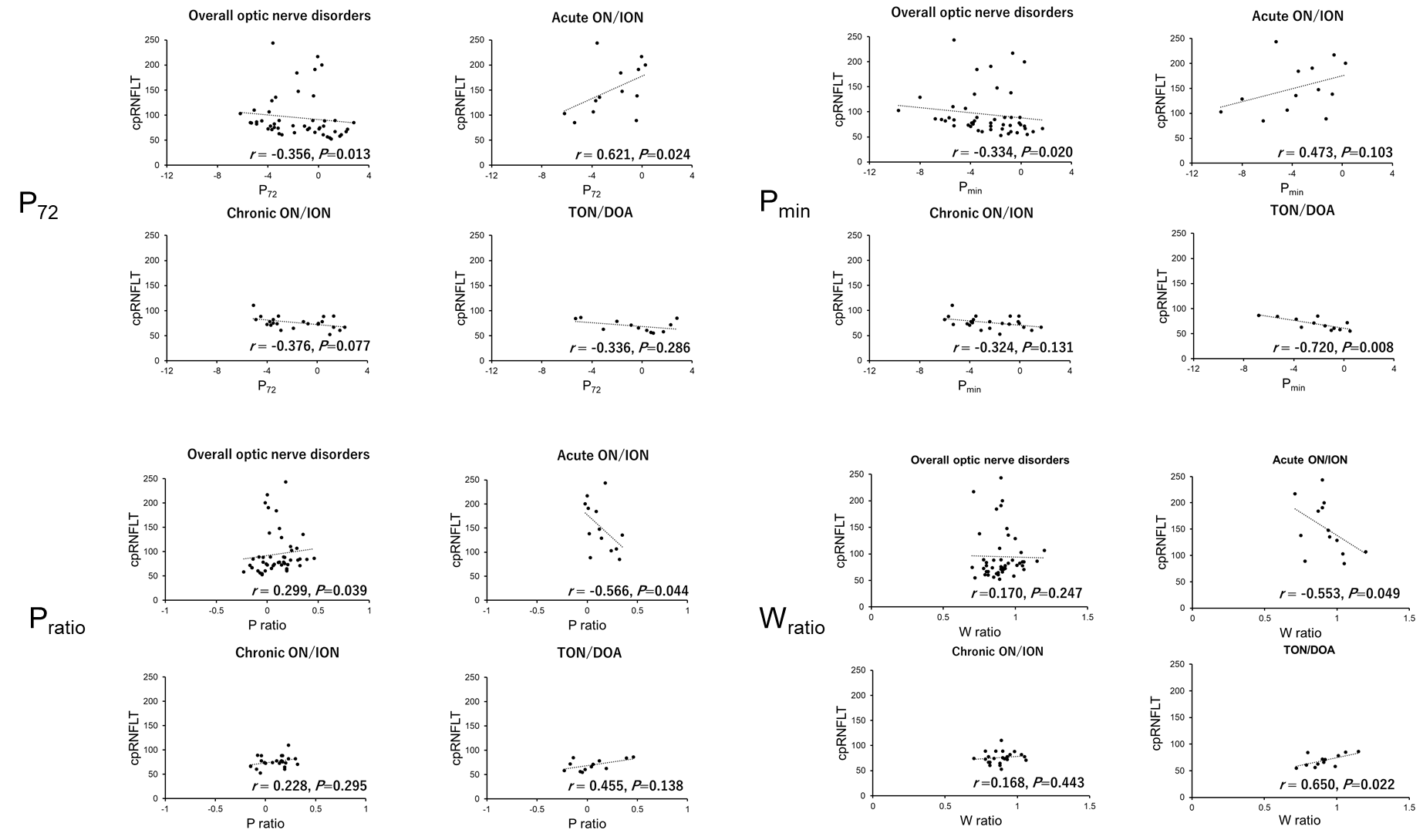
**

**Supplementary Figure 1. Graph showing the relationship between the PhNRs and cpRNFLT in optic nerve disorders.**

Scatterplots of PhNRs and cpRNFLT in overall optic nerve disorders, acute ON/ION, chronic ON/ION and TON/DOA. P72, upper left; Pmin; upper right; Pratio, lower left; Wratio, lower right.
